# Evaluation of AI-enhanced tele-ECG response time and diagnosis in acute chest pain patients

**DOI:** 10.3389/fcvm.2025.1532770

**Published:** 2025-11-20

**Authors:** Tarso Augusto Duenhas Accorsi, Fabio Grunspun Pitta, Juliane Rompkoski, Flavio Tocci Moreira, Renata Albaladejo Morbeck, Karen Francine Köhler, Karine De Amicis Lima, Carlos Henrique Sartorato Pedrotti

**Affiliations:** 1 Telemedicine Department, Hospital Israelita Albert Einstein, São Paulo, Brazil; 2 Cardiology, Hospital Israelita Albert Einstein, São Paulo, Brazil

**Keywords:** myocardial infarction, chest pain, telecardiology, electrocardiography, artificial intelligence

## Abstract

**Background:**

The impact of artificial intelligence in improving Tele-ECG response times and diagnostic accuracy among emergency patients experiencing acute chest pain remains uncertain. This study assesses the performance of AI-assisted cardiologists’ ECG report generation and characterizes diagnoses derived from examinations conducted at emergency facility without on-site cardiology services.

**Methods:**

A retrospective cross-sectional observational study at a Telemedicine Center in São Paulo, Brazil, examined ECG data from patients aged 18 and older with suspected ischemic syndromes at peripheral emergency departments in Goiânia, Brazil. Seventeen cardiologists carefully evaluated ECGs, focusing on identifying critical diagnostic red flags. Advanced AI algorithms enabled the accurate measurement of electrocardiographic segments and intervals, improving the detection of abnormalities and deviations from standard parameters.

**Results:**

Out of 25,346 ECG tracings submitted, 22,159 (87.42%) were analyzed. Unanalyzed tracings included 953 (3.75%) with artifacts, 633 (2.49%) with atrial fibrillation, 506 (1.99%) with inverted leads, and 628 (2.47%) with flat lines. The median age of patients was 49 (30–64) years, with 12,082 (54.52%) females. ST-segment elevation myocardial infarction (STEMI) was diagnosed in 202 (0.9%) cases. Other diagnoses included normal tracings, diffuse ventricular repolarization changes, sinus tachycardia, complete branch block, left ventricular hypertrophy, intraventricular conduction disorders, electrically inactive areas, sinus bradycardia, and atrioventricular conduction disorders. Request times averaged 11:30 AM (±7.07 h). The median response time was 75 (50–125) seconds, with a median of 375 (207–655) seconds for STEMI reports.

**Conclusion:**

Most ECGs are interpretable, but clearer tracings are needed. Quick response times are likely due to early AI detection of abnormalities. The low occurrence of acute myocardial infarction and other prognostic indicators suggests a low-risk group using the emergency department as their main healthcare access point.

## 1 Introduction

Acute ST-segment elevation myocardial infarction (STEMI) represents a significant portion of cardiovascular emergencies in Brazil, with reported in-hospital mortality rates reaching 17%. About half of these cases receive reperfusion therapy, mainly through fibrinolysis (1). Despite guideline recommendations, disparities in access to reperfusion treatment continue, especially in resource-limited settings (2). Telemedicine strategies, including tele-ECG, offer timely diagnosis and facilitate early intervention, improving treatment metrics across geographically diverse populations (3). A large tele-ECG study conducted by three Telemedicine (TM) Centers supporting 355 small clinics and primary health care centers in remote locations across four South American countries analyzed 780,234 patients. Telecardiology diagnosed 8,395 (1.1%) cases of STEMI, of which 3,872 (46.1%) were urgently treated at 47 hubs. Among these patients, 3,015 (78%) received reperfusion therapy via percutaneous coronary intervention. The average time-to-TM diagnosis was 3.5 min. During the study period, the average door-to-balloon time improved from 120 to 48 min, and the overall STEMI mortality rate was 5.2% (4). This benefit is evident in both urban and rural centers, reinforcing the role of TM with emergency electrocardiographic interpretation as a crucial resource within the healthcare system (5).

Although there is significant evidence supporting the cost-effectiveness of TM, its implementation continues to be difficult in developing countries (6). Artificial intelligence (AI) has many uses in cardiology, especially in electrocardiographic interpretation, supported by several promising studies with the potential to improve the use of TM strategies (7). Regarding STEMI, studies have shown that AI can diagnose the condition with accuracy comparable to that of a cardiologist (8). AI software with multi-labeling features to detect STEMI and other abnormalities can be used to help screen patients with suspected ischemic conditions (9). Recently, a randomized study demonstrated that AI-assisted ECG triage significantly reduced door-to-balloon time in STEMI patients at the emergency department (82.0 min vs. 96.0 min, *p* = 0.002) and ECG-to-balloon time in both emergency and inpatient settings compared with standard care (10).

The impact of AI on enhancing Tele-ECG response times and the differential diagnoses attained through this methodology in patients presenting with acute chest pain in emergency departments remains insufficiently understood. Consequently, this study aims to analyze the response time for generating ECG reports by cardiologists utilizing AI and to delineate the diagnoses obtained from examinations ordered in emergency facilities that are not referenced in cardiology.

## 2 Methods

### 2.1 Study design and participants

We conducted a single-center retrospective study at the TM Center of Hospital Israelita Albert Einstein in São Paulo, Brazil. The study protocol, referred to as the “TeleSUPRA” trial, along with the waiver of consent (based on the analysis of anonymized retrospective routine care data), was approved by the Review Board of Hospital Israelita Albert Einstein (registration number CAAE 76865723.4.0000.0071).

The TM Center, which coordinated the study, conducted all analyses. All authors unanimously agreed to submit the article for publication, affirming the integrity and accuracy of the data and the adherence of the trial to the established protocol.

Between August 2023 and March 2024, this observational and retrospective study utilized anonymized data from patients admitted to emergency rooms at six peripheral health facilities in Goiânia, Brazil. Analyses were conducted by a Telemedicine Center in São Paulo, where seventeen cardiologists utilized AI software to aid in identifying abnormal electrocardiogram patterns. A priority alert was issued for generating a report when the electrocardiogram exhibited ST-segment changes indicative of acute coronary syndrome. Consequently, cardiologists generated a corresponding report when the electrocardiogram changes satisfied the criteria for STEMI. They activated an alert, initiating a care protocol to transfer the patient to a specialized cardiology hospital for primary coronary angioplasty within 120 min. Each received ECG was mandated to be reported within 10 min. Furthermore, the AI software assisted in verifying PR, QRS, and QT interval measurements and identifying P and T waves, thereby detecting additional ECG changes pertinent to differential diagnoses for chest pain and supporting physicians in secondary hospitals in clinical decision-making. The study included adult patients (>18 years) presenting with suspected ischemic syndromes, for whom tele-ECG was requested by local teams for immediate reporting. Elective cases were excluded, and electrocardiographic reports emphasized primary findings.

The study size was determined based on the availability of ECG tracings submitted to the Telemedicine Center during the study period. A total of 25,346 ECG tracings were collected from six peripheral emergency health facilities; of these, 22,159 (87.42%) were analyzed. We excluded 3,187 (12.58%) ECG tracings that were deemed non-analyzable. These exclusions occurred due to the presence of artifacts (3.75%), atrial fibrillation (2.49%), inverted leads (1.99%), or flat lines (2.47%). These technical issues made it impossible to generate accurate diagnostic reports, so these cases were not included in the final analysis. No other forms of non-participation, such as patient refusal or drop-out, were applicable since the study relied on retrospective, anonymized data.

The inclusion of this sample size enabled a comprehensive evaluation of the impact of AI-assisted analysis in a real-world setting, ensuring that the data were both representative and statistically robust. The study's retrospective nature meant that the sample size was not predetermined but was based on the volume of routine clinical cases handled by the center during the specified period. The many ECGs analyzed provided sufficient power to detect significant outcomes, especially in less common conditions such as ST-segment elevation myocardial infarction (STEMI).

Several strategies were used to improve data integrity and objectivity in this study. First, all patient data were anonymized, minimizing the risk of subjective bias related to patient demographics or clinical history. This ensured that interpretations relied solely on the electrocardiogram (ECG) data rather than external factors.

The data were collected from multiple peripheral health facilities, which helped improve the generalizability of the findings and reduce location-based biases. The ECG data were obtained using iMedECG 12-lead digital electrocardiograph machines, standardized at 25 mm/sec and 10 mm/mV. Each ECG was sent to a centralized cloud server for AI-assisted interpretation and analysis. The AI model does not include clinical metadata (e.g., age, symptoms) but strictly assesses waveform morphology. Cardiologists used AI-augmented cues to prioritize and validate reports. Only certified cardiologists performed the final interpretation. Each ECG was reviewed by a single cardiologist. Additionally, using AI software to assist with ECG analysis provided a standardized approach, decreasing variability and human error. The AI system was designed to reliably identify key abnormalities. Meanwhile, the final interpretation and report generation were still carried out by cardiologists, enabling cross-validation and reducing reliance solely on AI-driven results.

Furthermore, ECGs with technical problems, such as artifacts or inverted leads, were excluded from the analysis to avoid bias from non-diagnostic data. Additionally, since the study was retrospective, the data reflected routine clinical practice, reducing the risk of selection bias. These measures together enhanced the reliability of the study's findings and minimized potential sources of bias.

### 2.2 Artificial intelligence model specification

The AI algorithm used in this study is a convolutional neural network (CNN) developed and trained on a proprietary dataset with over one million expert-annotated 12-lead ECG recordings. The model was designed as a multi-label classifier capable of detecting a wide range of electrocardiographic abnormalities, including but not limited to STEMI, conduction disorders, and repolarization abnormalities.

The CNN architecture includes multiple convolutional layers optimized for extracting temporal and morphological features from raw ECG waveform data. The model processes standardized digital inputs at a 500 Hz sampling rate and provides diagnostic probabilities for each label, along with segmentations of the P, QRS, and T waves. Internal validation showed an area under the receiver operating characteristic curve (AUROC) of 0.98 for STEMI detection, with an overall diagnostic accuracy exceeding 91%.

Model inference runs on a secure cloud infrastructure. When an ECG is transmitted, the AI system performs automated segmentation and interval measurements (PR, QRS, QT), flags abnormal findings, and produces a structured preliminary analysis. This output is then accessible to the on-call cardiologist through the telemedicine platform. While the AI assists in prioritization and supports interpretation, the final diagnostic report is exclusively written by board-certified cardiologists, ensuring clinical oversight.

This AI-powered workflow was created to enhance triage efficiency, especially in settings without immediate cardiology expertise, and to support real-time decision-making in emergencies.

**Figure 1:**
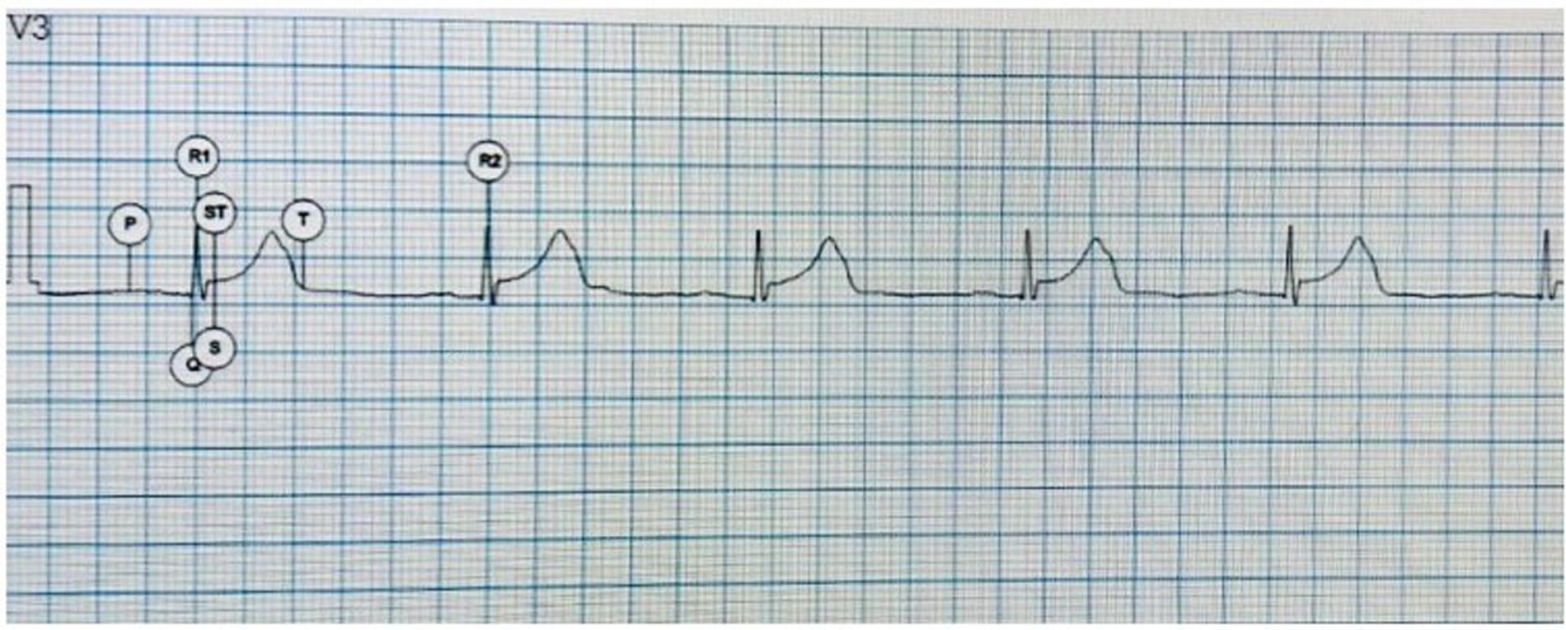
Electrocardiographic waves identified by AI. P, P wave; Q, first negative wave of the QRS complex; R1, first positive wave of the QRS complex; S, negative second wave of the QRS complex; ST, ST and T segment, T wave.

The software quickly analyzes the trace and recognizes the primary waves, placing the respective letters (Figure 1).

**Figure 2:**
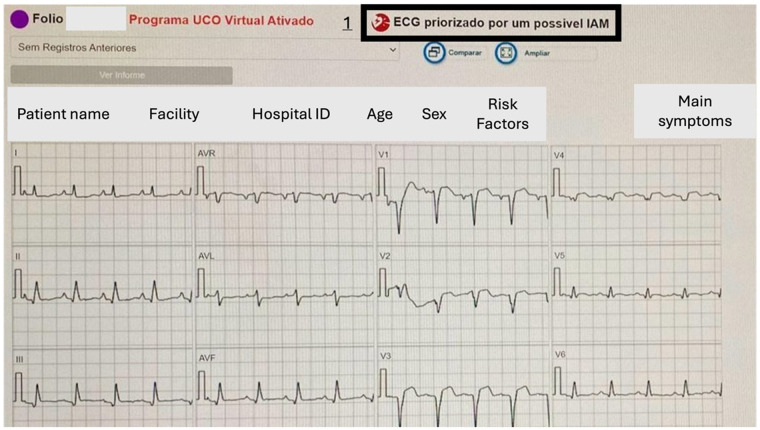
ST segment elevation identified by a disparity in levels between the T and PR segments indicates a potentially abnormal ECG signal. Number 1 points to the warning box for possible STEMI: in Portuguese, “ECG prioritized for a possible AMI.” Above the ECG are the data received by the telecardiology.

The software also recognizes deviations in the ST segment and alerts you to the possibility of STEMI (Figure 2).

### 2.3 Primary outcomes

The study aimed to evaluate the turnaround time for ECG report generation by cardiologists using AI and to describe the diagnoses resulting from examinations requested at non-specialized emergency facilities in cardiology.

### 2.4 Data extraction

All data was collected anonymously from the project database. Notably, the TM Center only stored data related to the ECG and the time taken to generate the report. Some clinical variables, such as patient symptoms and risk factors, were available and analyzed alongside the ECG to produce the report. However, these variables were not studied in detail because they were entered manually by nursing staff into the ECG system and were not available electronically for analysis.

### 2.5 Statistical analysis

The statistical analysis in this study was mainly descriptive and aimed to summarize important variables related to the ECG tracings and diagnostic outcomes. Categorical variables were shown as counts and percentages, while continuous variables were given as means with standard deviations for data that followed a normal distribution, and medians with interquartile ranges for data that did not. This method provided a clear picture of the data without assuming any specific distribution. Statistical analysis was conducted using IBM SPSS Statistics for Windows, version 22.0.

Because the study is retrospective and observational, no specific statistical methods were used to control for confounding factors. The focus was on reporting outcomes based on the available data, and no adjustments or multivariable analyses were conducted to account for potential confounders like patient comorbidities or other clinical factors. As a result, while the findings provide useful insights, they reflect observational data and should be interpreted accordingly.

The final analysis of this study did not include any missing data. All data, including ECG tracings and related diagnostic outcomes, were complete. Cases with unusable ECG tracings due to artifacts, inverted leads, or flat lines were excluded from the analysis. These exclusions were considered non-analyzable rather than missing data, ensuring the final dataset only contained viable tracings for diagnostic interpretation. Therefore, there was no need for specific handling or imputation methods for missing data.

No specific sensitivity analyses were performed in this study. The research mostly involved descriptive statistical analysis of ECG tracings and diagnostic outcomes without using methods to test how robust the results are under different scenarios. Sensitivity analyses were unnecessary due to the retrospective design and complete data. The exclusion of unusable ECGs, such as those with artifacts or inverted leads, was consistent throughout the analysis, ensuring the dataset remained standardized and no additional sensitivity adjustments were needed.

## 3 Results

From 25,346 electrocardiographic submitted tracings, 22,159 (87.42%) were analyzed. No participants had missing data for the primary variables of interest in this study. All data related to the electrocardiograms (ECGs) and their respective diagnostic outcomes were fully available. Cases where ECGs could not be analyzed due to technical issues were excluded from the analysis. Among the unanalyzed tracings, 953 (3.75%) contained artifacts, 633 (2.49%) showed atrial fibrillation, making analysis impossible, 506 (1.99%) had inverted leads, and 628 (2.47%) presented a flat line. Therefore, no missing data were reported for the remaining variables of interest, as the analysis included only those ECG tracings that were viable for interpretation.

In this cross-sectional study, 25,346 ECG tracings were initially submitted for analysis, of which 22,159 (87.42%) were successfully analyzed. The primary outcome of interest was the identification of ST-segment elevation myocardial infarction (STEMI), which occurred in 202 cases, representing 0.9% of the total ECGs analyzed. Other key diagnostic outcomes included normal ECG tracings in 44.25% of cases, diffuse ventricular repolarization changes in 21.92%, sinus tachycardia in 9.32%, complete branch block in 4.56%, left ventricular hypertrophy in 4.34%, and various other conduction or repolarization abnormalities. The median response time for ECG reports was 75 s overall, with a median of 375 s for STEMI cases.

All estimates in this study were unadjusted, and no confounder-adjusted estimates were used. The analysis concentrated on descriptive statistics, reporting outcome measures such as the incidence of STEMI and other ECG findings. Because the study did not include multivariable analysis or adjustments for potential confounders, no confidence intervals or precision estimates (e.g., 95% CI) were calculated. The study's descriptive approach aimed to provide a straightforward summary of diagnostic outcomes and reporting times without adjusting for confounding variables. Consequently, no confounding variables were identified or controlled for in this analysis.

In this study, the main continuous variable that was categorized was the patients’ age. Age was treated as a constant in descriptive statistics, but no specific categorization or binning was applied to it for analysis. Other continuous variables, such as ECG report times, were reported using medians and interquartile ranges but were not divided into discrete groups. Therefore, no boundaries for categorizing continuous variables were provided.

**Figure 3:**
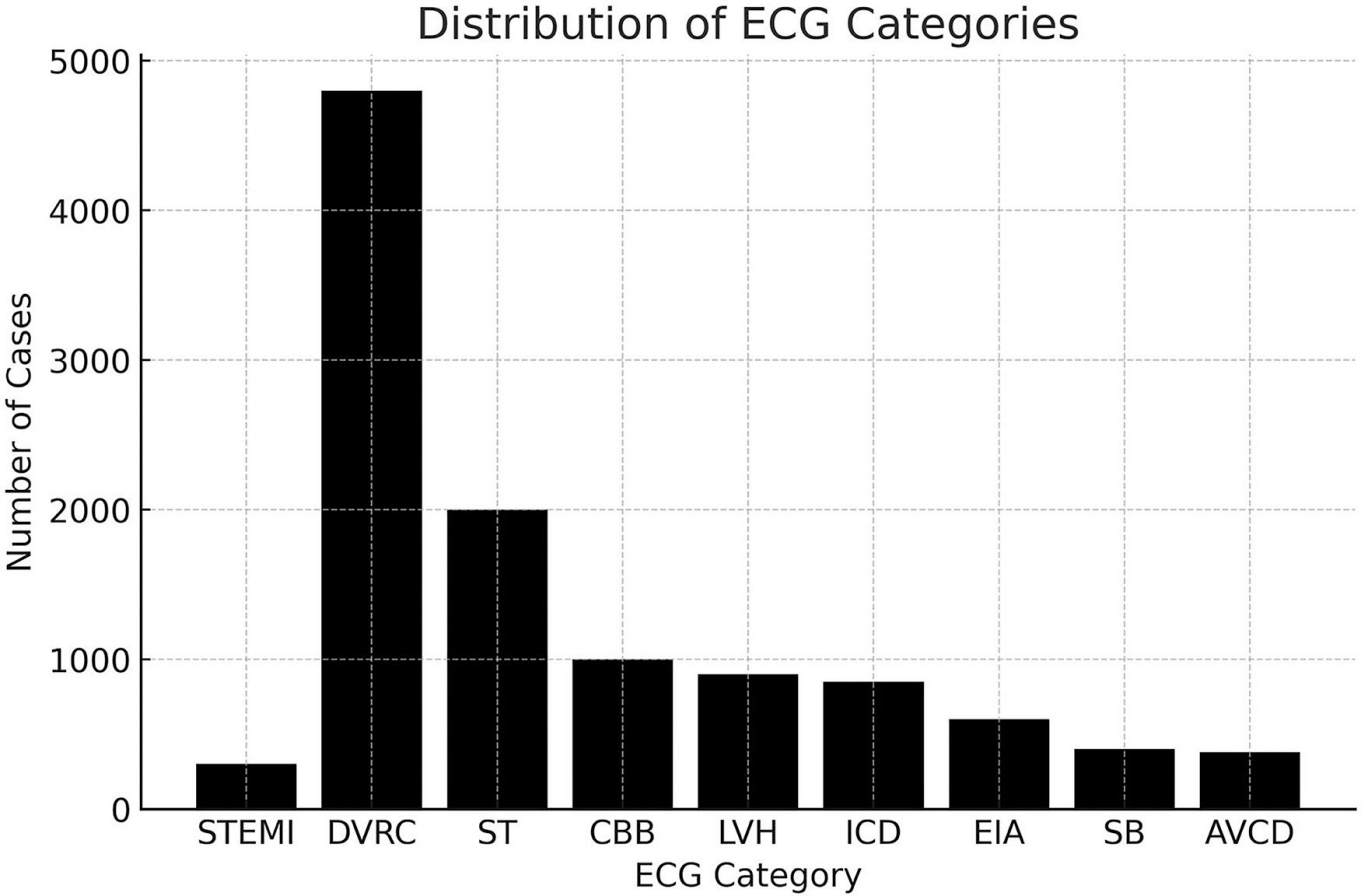
The primary diagnosis provided by AI-enhanced TeleECG. AVCD, atrioventricular conduction disorders; CBB, complete branch block; DVRC, diffuse ventricular repolarization changes; EIA, electrically inactive area; ICD, intraventricular conduction disorders; LVH, left ventricular hypertrophy; SB, sinus bradycardia; ST, sinus tachycardia; STEMI, ST-segment elevation myocardial infarction.

The median age was 49, interquartile range (IQR) of 30–64 years, and 12,082 (54.52%) were female. These distinct demographic data were derived from the analysis of the electrocardiographic tracings. There were 202 (0.9%) cases of ST-segment elevation myocardial infarction (STEMI) diagnosed. (Figure 2) Other diagnoses included 9,806 (44.25%) normal tracings, 4,857 (21.92%) diffuse ventricular repolarization changes, 2,065 (9.32%) sinus tachycardia, 1,011 (4.56%) complete branch block, 962 (4.34%) left ventricular hypertrophy, 859 (3.88%) intraventricular conduction disorders, 583 (2,63%) electrically inactive area, 428 (1,93%) sinus bradycardia, 416 (1.88%) atrioventricular conduction disorders (Figure 3).

**Figure 4:**
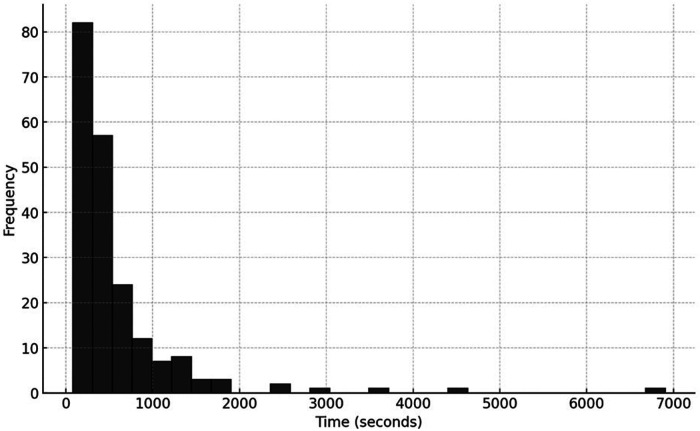
Distribution of report issuance time.

The distribution of request times was normal, with an average at 11:30 AM (±7.07 h). The median response time was 75 (50–125) s. In STEMI cases, the team sent the report within a median of 375 (207–655) s (Figure 4).

In this study, no additional analyses, such as subgroup, interaction, or sensitivity analyses, were performed. The focus stayed on descriptive statistical reporting of the overall population without breaking it down into specific subgroups or examining interaction effects. The lack of multivariable or subgroup analysis matched the retrospective observational design, which aimed to evaluate AI-assisted ECG analysis in a broad population context. As previously stated, no sensitivity analyses were conducted since the study mainly used complete and analyzable data.

## 4 Discussion

Most TeleECG studies have concentrated on evaluating standard quality metrics in STEMI, such as door-to-needle and door-to-balloon times, beyond mortality. However, the exact duration for report issuance has been relatively understudied (11). Our study showed that the issuance of reports by cardiologists, which detail the primary ECG diagnosis via TM, was significantly sped up by software capable of detecting deviations from the norm. In a dataset of over 20,000 reports, the average issuance time was impressively low, at just 75 s.

We highlight that AI facilitated not only prioritization but also significant decreases in cardiologist response times. An overall median of 75 s and 375 s for STEMI cases demonstrate strong performance in settings without onsite cardiologists. The longer review times for STEMI cases were due to the need for diagnostic caution before activating limited catheterization resources.

A recent study addressed the duration required to issue electrocardiographic reports via TM. Even with the use of a push notification system, the issuance time ranged from 5.75 ± 1.63 min (12). In the pre-hospital setting, mobile intensive care ambulances experienced an increase of approximately 3.7 min when transmitting the electrocardiogram. However, this did not significantly impact the average transport time to the hospital (13, 14). Substantial evidence supports the benefit of early ECG transmission for specialized analysis in pre-hospital care, particularly when patients are attended to by paramedics or nurses (15). The primary objective of rapid electrocardiographic analysis in the pre-hospital scenario is to promptly activate the system that enables cardiac catheterization, facilitating timely primary angioplasty or, if unavailable, to anticipate fibrinolysis (16). This present study analyzed the support provided by TM in the Tele ECG modality for patients who spontaneously seek secondary emergency facilities. These services serve as a reference for large populations in developing countries and frequently suffer from a shortage of specialists and resources while simultaneously presenting high demand for medical care (17). Some supporting evidence shows that TM enhances quality and care metrics in these units, similarly to its impact on pre-hospital care (18). However, the impact of TeleECG in this context has been underexplored, motivating the analysis conducted in this study.

Integrating AI into TM platforms, particularly for ECG analysis, represents a significant advancement in managing patients presenting with chest pain. AI can recognize deviations from normal in electrocardiographic monitoring (19). A review of 15 studies reported misdiagnosis rates of approximately 1%–2% for STEMI, with higher rates observed in non-specialized hospitals (20). Conversely, there is substantial evidence indicating that software designed to detect deviations from normal electrocardiographic patterns possesses a high negative predictive value, which has the potential to minimize diagnostic errors in cases of STEMI (21). This study underscores the pivotal role of AI-enhanced TeleECG in improving the issuance time of specialized reports and in diagnostic accuracy, thereby enhancing the clinical decision-making process in settings often characterized by limited access to specialized cardiology expertise. Our findings indicate that implementing AI in TeleECG is related to low report time, essential for improving patient outcomes. This expedited process enhances the quality of care and optimizes workflow within emergency departments, allowing for better allocation of medical resources.

Although our study did not include a comparator arm without AI support, the response times observed in our cohort are significantly shorter than those reported in previous telecardiology studies that relied solely on manual ECG interpretation by cardiologists.12 While we acknowledge that the absence of a control group limits direct causal inference, these findings align with prior evidence showing that AI integration in ECG interpretation workflows can speed up diagnostic processes, especially by highlighting critical abnormalities that need urgent attention. Moreover, existing literature indicates that general practitioners and emergency physicians, particularly in resource-limited or non-specialist environments, may have lower sensitivity and longer response times when identifying life-threatening electrocardiographic changes such as STEMI. Therefore, the AI tool used in this study likely served two complementary roles: (1) accelerating cardiologists’ focus toward high-priority tracings through automated alerts, and (2) providing standardized interval measurements and waveform segmentation to enable rapid report creation. These mechanisms could be especially valuable in remote emergency units without on-site cardiologists, where delays in diagnosis could directly impact patient outcomes. We recognize that a randomized comparison between AI-assisted and unaided cardiologist workflows would provide stronger evidence. Nevertheless, our real-world results support the growing body of literature suggesting that AI-enhanced tele-ECG platforms offer both diagnostic support and operational advantages in acute care settings.

Despite the low average reporting time, we found that the median reporting time for cases of STEMI was 375 s. The face-to-face services targeted in this study were recently organized into a network with substantial government investment. We attribute the longer notification time in cases of STEMI to the fact that MT cardiologists carried out an analysis and discussion looking for differential diagnoses for cases suggestive of STEMI, as they only had the electrocardiographic tracing and some relevant clinical data to establish the diagnosis. This careful consideration is aimed at ensuring the judicious use of the limited resources available for activating the hemodynamics service. However, this time is comparable to those found in studies conducted in large centers (22–24).

Approximately 12% of the tracings were initially challenging to read due to artifacts, atrial fibrillation, or lead-related issues. This initial data exceeds the problems reported in real-life transmission studies (25). We did not account for or analyze subsequent requests for interpretation arising from new tracings for the same patient under treatment. Even with computer transmission in a hospital environment, we encountered more artifacts that rendered analysis impossible compared to studies utilizing simpler technologies, such as mobile phone transmission, even in pre-hospital settings (26). These data underscore the pressing need for training the local health team in secondary emergency facilities to optimize the quality of transmitted tracings. Enhancing the quality of transmissions is essential for TeleECG to achieve the goals proposed by the ACC/AHA for the treatment of STEMI (27). This is particularly important because the primary beneficiaries are elderly individuals, residents of remote areas, and those whose primary point of care is secondary hospitals (28).

In our study, 0.9% of patients were diagnosed with STEMI, a prevalence comparable to other TeleECG studies (3, 4). Virtually all scientific evidence regarding AI-powered ECG analysis is focused on STEMI (29). Its implementation is associated with a reduction in the activation time of the STEMI protocol and an overall improved prognosis (8, 30, 31). The accuracy of AI is comparable to that of cardiologist interpretations and often surpasses the analysis conducted by other specialties (9, 32). Despite these promising advancements, the impact of TeleECG in secondary emergency units remains underexplored. Our study provides critical insights into AI-assisted ECG analysis's operational benefits and clinical implications in such contexts. Future research should focus on longitudinal outcomes and the cost-effectiveness of integrating AI into telemedicine frameworks, particularly in underserved regions.

Regarding the differential diagnoses issued, there are no previous studies that have addressed this analysis using AI. Similarly, there is a lack of clarity in the diagnoses issued by TeleECG. Our cardiologists identified 44.25% of the tracings as normal, with the primary differential diagnosis being diffuse changes in ventricular repolarization, observed in 21.92% of cases. Less than 10% of patients exhibited changes strongly suggestive of structural heart disease that could account for their symptoms. These data suggest that most patients are at low risk, which also facilitates the rapid issuance of reports. A Telemedicine Center with a cardiologist available 24/7, equipped with an alert system for incoming ECGs, also proved to be essential for rapid reporting and resolution of patients with STEMI.

Notably, technical limitations such as poor-quality ECG tracings remain a significant challenge. While staff education is critical, we acknowledge the need for infrastructure improvements, including device maintenance, shielding against electromagnetic interference, and robust digital transmission systems.

Regarding Brazil's readiness for broader TeleECG adoption, wearable and mobile ECG solutions are not yet widely integrated in public health services, particularly in rural areas. Additionally, most peripheral emergency services lack comprehensive electronic medical record (EMR) systems, posing challenges for interoperability and real-time data linkage.

Feedback from participating cardiologists indicated high satisfaction with AI as a support tool. However, human oversight was consistently emphasized due to the occasional occurrence of false positives. No formal user satisfaction survey was conducted, which represents a limitation that we plan to address in future work.

### 4.1 Limitations

This study has limitations. It did not include measurements or availability of clinical history and physical examination data, focusing mainly on the electrocardiographic tracing; it also lacked measurements of STEMI care metrics; no comparisons between groups were made; and the AI software provided diagnostic suggestions by detecting deviations from the norm, which always required a final report from a cardiologist. Furthermore, this study did not incorporate clinical metadata (e.g., full history, physical exam) or longitudinal outcome data. Additionally, inter-rater variability and satisfaction with AI were not formally assessed, and there was no external validation of the AI model in this cohort. The AI system offered interpretative suggestions but was not autonomous; a human cardiologist always made the final diagnosis.

## 5 Conclusion

The study shows that most electrocardiograms are analyzable, but more interpretable tracings are needed. Quick response times probably result from initial AI analysis detecting deviations. The low incidence of acute myocardial infarction and other prognostic changes indicate a low-risk population using the emergency department as their primary healthcare access point.

## Data Availability

The raw data supporting the conclusions of this article will be made available by the authors, without undue reservation.
